# A three-years follow-up of extra intensive support for individuals with intellectual disability and severe challenging behaviour in the Netherlands

**DOI:** 10.1177/17446295241252918

**Published:** 2024-05-09

**Authors:** Linda Verhaar, Yvette M. Dijkxhoorn, Leo de Sonneville, Hanna Swaab

**Affiliations:** SCORE project: Ipse de Bruggen, De Hartekamp Groep, Cordaan and Ons Tweede Thuis, the Netherlands; Clinical Neurodevelopmental Sciences, 4496 Leiden University, The Netherlands; Clinical Neurodevelopmental Sciences, 4496 Leiden University, The Netherlands; Clinical Neurodevelopmental Sciences, 4496 Leiden University, The Netherlands; Leiden Institute for Brain and Cognition, The Netherlands

**Keywords:** adaptive behaviour, challenging behaviour, intellectual disability, intensive support needs, quality of life

## Abstract

Adults with Intellectual Disability who show severe challenging behaviour need intensive individual support. If intensive support proves to be insufficient, extra intensive support can be provided in the Netherlands, which is characterized by more time for individual care. The present study evaluates the impact of extra intensive support over time. Client characteristics of adults receiving intensive support (IS, *N*=70) or extra intensive support (IS+, *N*=35) are compared and the impact of provided support on challenging behaviour (Developmental Behaviour Checklist-Adults), adaptive behaviour (Vineland II), and Quality of Life (San Martin Scale) is evaluated over a three years period. Compared to adults receiving intensive support, those receiving extra intensive support initially showed higher intensity of challenging behaviour, higher number of mental health diagnoses and stronger focus on goals to reduce challenging behaviour. Over time, intensity of challenging behaviour decreased in adults receiving extra intensive support, although Quality of Life and adaptive functioning did not improve. Results show that the indications for receiving extra intensive support are clear and that the extra support is effective over time. It is concluded that extra individual support is serving those who need this support.

## Introduction

Individuals with intellectual disability show a large etiological heterogeneity (Burack et al., 2021). Due to this variability, individuals with intellectual disability and challenging behavior (CB) show a wide range of support needs, defined as the range and intensity of supportive interventions necessary to attend to daily essentials, social activities, learning and working (Thompson et al., 2009). In addition to support associated with specific medical and behavioural problems, support needs arise from a combination of an individual’s skills, demands and complexity of settings and activities he or she must participate in (Thompson et al., 2009). Besides the evaluation of cognitive functioning (IQ), adaptive behaviour, health, participation, context and their reciprocity, according to the model of human functioning by the American Association of Intellectual and Developmental Disabilities (AAIDD-model) (Schalock et al., 2021), an integrative diagnosis of an individual with intellectual disability should also include an analysis of individual support needs, foremost to optimize the effectiveness of tailor-made support plans, living arrangements and resource allocation.

In the Netherlands, approximately 13% (CBS, 2023) of the population of persons with intellectual disability receiving long-term care is presenting with severe challenging behaviour, needing 24 hour Intensive Support (IS). Support needs are based on diagnostic evaluation, and reflect the individual’s personal needs that should be timely and regularly monitored and evaluated (Buntinx and Schalock, 2010b; Schalock et al., 2021). Support is strongly focused on improving quality of life (QoL), which is defined as a set of domains of functioning determining a person’s well-being, the outcome being a combination of factors pertaining to physical, emotional and material wellbeing, self-determination, rights, personal development, social inclusion and interpersonal relationships (Schalock et al., 2002). Challenging behaviour is a well-known risk factor associated with lower quality of life (Emerson, 2001; Robertson et al., 2004). Challenging behaviour is defined by Emerson (2001) as behaviour which is culturally abnormal, endangers the physical safety of the person (or others), or limits the use/access to ordinary community facilities. People with intellectual disability are more at risk for challenging behaviour and related restrictive measures (Allen et al., 2009), social exclusion, and lack of autonomy (Griffith et al., 2013), that inevitably and unfavourably influence quality of Life.

Lower levels of cognitive functioning and adaptive behaviour have been linked to more severe challenging behaviour in people with intellectual disability (Bowring et al., 2019; Felce et al., 2009). The presence of challenging behaviour relates to a higher need for behavioural support in general (Seo et al., 2017) and for social support and maintenance of emotional well-being in particular (Lamoureux-Hebert et al., 2010). Bruinsma et al. (2022) found that for support staff higher educational level, friendly behaviour and more self-efficacy, i.e. the feeling of confidence and satisfaction in being able to deal with challenging behaviour is associated with higher quality of life in the individual with intellectual disability and challenging behaviour. 

When the level of intensive support does not suffice in meeting a person’s support needs, an even higher level of support can be provided in the Netherlands after applying successfully for extraordinary funding. This extraordinary funding enables to provide extra Intensive Support (or IS+). This highest level of support becomes available when the challenging behaviour is not understood, is considered too intense or resistant to current supportive regulation. When it is decided that these individuals are entitled to this additional care, they receive the highest level of intensive support (IS+), through extra financial means. These means are mainly aimed at increasing direct care involvement, by allowing more time to be invested, resulting in a higher client-direct staff ratio, often reaching 1:1 for a significant portion of the day or the whole day. Through an increased staff presence, it is thought that there is more real-time, proactive and therefore effective responsiveness to the individuals’ extra intensive support needs due to their challenging behaviour.

In the Netherlands, funding of extra intensive support ultimately aims at regaining quality of life within the setting of one’s own (group)home, avoiding hospitalization and segregation. Funding is provided by the government and is allocated through a system in which expert clinical judgement is decisive (ZorgverzekeraarsNederland, 2023). These extra resources are received for a certain period of life, as long as deemed necessary, based on repeated clinical judgement. Nationwide, the number of clients for whom this funding has been applied for, has increased enormously in the last few years (Hopman, 2021), due to different factors. Shifting towards more humane support, restrictive measures are no longer viewed as acceptable (Lawrence et al., 2022). The present paper aims to explore efficacy of extra intensive support. Furthermore, since decision criteria for extra intensive support are potentially subjective, this study aims to contribute to improve the assessment framework of allocation of financial resources for extra intensive support and the development of guidelines for clinical evaluation of the effect of extra intensive support .

Outside the Netherlands, this extra intensive support group may be regarded somewhat comparable to ‘out-of-area’ placements of people with intellectual disability and complex mental health needs in England, and, for example, people with intellectual disability and severe challenging behaviour residing in small group homes in the USA (Tevis, 2020). As a result of de-institutionalization policies, specialistic settings for complex cases in England are sparsely located. These hospitals are considered essential for highly specialistic care when severe challenging behaviour is present (Barron et al., 2011; Bonell et al., 2011; Deveau et al., 2015).

Tevis (2020) summarized what factors were most relevant in service costs for people with intellectual disability and severe challenging behaviour residing in small group homes (1-3 persons, 24/7 care, Nebraska USA). Some of these people receive extra ‘exception funding’, approved by state officials, in which eligibility criteria are often mostly qualitative, inconsistent and subjective (Tevis, 2020), which situation reflects seemingly similar shortcomings present in the Dutch application system for extra intensive support funding.

Higher support needs are associated with higher costs by the addition of extra staff, and, for example, also staff related costs like extra supervision, training of aggression prevention, and management training. Tevis (2020) found that the combined impact of comorbid mental and physical health problems and aggressive behaviour contributed significantly to higher service costs. More specifically, the frequency and severity of aggressive behaviour contributed more to the rise in costs than the level of cognitive and adaptive dysfunctioning. Management of aggressive behaviour requires more direct support throughout the organization and staff training, but also more indirect support, such as clinical oversight, increased and continuous monitoring (Tevis, 2020).

In the Netherlands, few studies have been conducted regarding the characteristics and efficacy of these extra intensive support. Claes et al. (2009) found that lower adaptive functioning was associated with higher support needs in a general intellectual disability population. Sandjojo et al. (2018) found that self-management training of people with moderate to mild intellectual disability decreased their support needs. To our knowledge, previous studies on clinical characteristics of individuals needing extra intensive support are nonexistent for the Dutch population.

The current study aims to follow-up individuals receiving intensive support or extra intensive support over a three year period in order to explore efficacy of intensive support and extra intensive support. Furthermore, the study intends to identify factors that are instrumental to distinguish between individuals entitled to intensive support and extra intensive support, i.e. level and characteristics of challenging behaviour, adaptive behaviour, quality of life, diagnostic classifications and chosen goals in individual support plans, taking into account relevant previously studied factors, i.e. gender, age and cognitive functioning (Unwin et al., 2017; Beadle-Brown et al., 2006; Wehmeyer et al., 2009; Knapp et al., 2005; Deveau et al., 2015). The extra intensive support group is expected to be characterized by a combination of severe challenging behaviour *and* lower adaptive behaviour *and* lower quality of life, compared to the regular high intensive support group. Further, differences in individual support plans and number of mental health classifications will be explored. In line with Hassiotis et al. (2008), a higher number of mental health diagnoses in the extra intensive support group is expected. Furthermore, it is expected that the focus of goals in Individual Support Plans (ISPs) will be on reducing challenging behaviour since aggression may be more prevalent in the extra intensive support group (Tevis, 2020). Lastly, the development of both groups on measures of challenging behaviour, adaptive behaviour and quality of life will be followed over time.

## Method

### Procedure

Data used in this study were collected during a large longitudinal project, SCORE project. The SCORE project evaluates the effects of regular care of persons with intellectual disability and intensive support need due to persistent and severe challenging behaviour (CB), living in staffed group homes. Care-as-usual was evaluated in four participating Dutch residential disability services for people with an intellectual disability [Ipse de Bruggen, de Hartekamp Groep, Cordaan and Ons Tweede Thuis].

Persons were eligible for inclusion when they met the following criteria: Adults with intellectual disability, living in staffed group home, needing (regular) intensive support or extra intensive support, due to persistent and severe challenging behaviour (CB), excluding those who needed intensive support primarily because of profound intellectual and multiple additional (neuromotor/sensory) disabilities. The participants were informed about the study by written and digital information. Most participants were unable to give *informed consent* themselves*,* therefore permission was obtained from legal representatives.

The study has a longitudinal design, collecting survey data in three time waves, starting in 2017 (Time 1, T1), with follow-ups in 2018 (Time 2, T2) and 2020 (Time 3, T3). Ethical approval was granted by the ethics committee of the of the Faculty of Social and Behavioural Sciences, Department of Clinical Neurodevelopmental Sciences, University of Leiden, The Netherlands (ECPW-2015/094), and the ethical committee of the largest participating residential disability service organization (Ipse de Bruggen).

*Dutch care system* In the Netherlands, participants with intensive support (IS) mainly reside in specialized staffed group homes, where they receive 24 hour care for support in all areas of life, living mainly in group homes varying in size. Extra intensive support (IS+) is available for those already receiving the most intensive regular support, through an independent eligibility process, based on qualitative assessment of psychological reports on a person’s functioning, individual support plan, and opinions of staff. In case an individual is eligible for IS+, as argued by the service organization, more financial means become available for the service organization to spend on support specifically for this individual. With these means the individuals can often remain in the same group home, which can be a mix of individuals receiving IS or IS+. Extra intensive support most often involves the availability of more time for individual direct support provided by caretakers, and can also result in environmental adjustments like extra individual facilities to support daily functioning. Typically, in intensive support the client-staff ratio in direct caretaking is 4/3:1, this can go up to 1:1 (or 1:2) for participants who receive IS+. Furthermore, more time for the multidisciplinary support team is available to intensify analysis of individual support needs. The multidisciplinary support team may include professionals like physicians, psychologists/orthopedagogues, occupational therapists.

### Participants

*N* = 152 participants receiving IS (*N* = 117) or IS+ (*N* = 35) were included at the first time wave. Participants’ age ranged from 18.02 years to 76.96 years (*M=*40.23, *SD=*14.22). For reasons of comparison with a group of participants receiving IS+, participants receiving IS were selected by person to person matching on level of cognitive functioning, age and gender. Two matches were selected for each participant with IS+, resulting in a comparison group of *N*=70 participants with IS.

In the longitudinal outcome analyses, 21 participants (8 IS+, 13 IS) were lost at the third time wave. It was analyzed with *t*-tests whether these differed from the participants remaining in the study, separately for the IS+ and the IS group. No differences were found for the IS+ group (.845*>p>*.229) nor for the IS group (.849*>p>*.117 ) on measures of CB, intellectual disability, adaptive behaviour, QoL, gender distribution or age.

### Measures

#### Level of cognitive functioning

For every participant, IQ scores were derived from scores on standardized tests, which were assessed within the last five years, before inclusion in the study, such as the Dutch versions of the Bayley Scales of Infant Development-II-NL (BSID-II-NL)/Bayley-III (Baar et al., 2014), Wechsler Intelligence Scale for Children-III_NL (WISC-III-NL), Wechsler Preschool and Primary Scale of Intelligence (WPPSI-III-NL), Wechsler Adult Intelligence Scale-III-NL (WAIS III-NL) (Wechsler, 1989; Wechsler, 1991; Wechsler, 2008; Kort et al., 2005; Wechsler, 2012), Snijders-Oomen nonverbal-revised (SON-R), Snijders-Oomen nonverbal test 2-8 - (Tellegen and Laros, 2011; Tellegen and Laros, 2017). These tests have sufficient reliability and validity. Variation in developmental/IQ tests was due to variation of use in clinical practice. Often in clinical practice among adults with intellectual disability in the Netherlands, intelligence/developmental tests available for a lower chronological age are used (Kraijer and Plas, 2014; Došen, 2014), to accommodate their relatively low level of cognitive functioning.

Participants’ own psychologist decided which assessment instrument was best suitable to determine each individuals’ profile, based on level of functioning (language use, motor skills etc.) and aim of assessment, in line with clinical guidelines (Embregts et al., 2019). To facilitate comparability and interpretation of scores, all outcomes were converted to cognitive age equivalents in months.

#### Challenging behaviour: Developmental behavioural checklist – Adult (DBC-A)

The DBC-A is a carer-completed 107-item questionnaire that assesses a comprehensive range of emotional, behavioural and mental health problems in adults with mild, moderate and more severe levels of intellectual disability (Mohr et al., 2005; Mohr et al., 2011; Mohr et al., 2012). Professional primary caretakers were asked to fill out the questionnaire, rating the answer to each item with never, sometimes or often/frequent. Six problem domains are distinguished, i.e. Disruptive behaviour, Communication and Anxiety Disturbances, Antisocial behaviour, Self-absorbed behaviour, Depressive and Problems Relating Social, and a Total score. Mean Item Score (MIS) of DBC-A Total problem behaviour was used as a measure of challenging behaviour (CB), i.e. DBC-A total (MIS). A higher score denotes more CB. Domain total scores of CB were also converted to mean item scores for a valid comparison between domains. Equivalent mean scores can either be derived by a large number checked ‘1’ or a low range of items checked ‘2’. To gain a better understanding of experienced intensity of behaviour, the DBC-A Intensity Index was calculated, i.e. the proportion of the positively checked items that were scored 2, as suggested by Taffe et al. (2008), by first determining the Proportion of Items checked for each participant (DBC-A PIC, total number of checked items/total items of questionnaire items). The sum of items checked 2 per participant was then divided by their PIC, resulting in the DBC-A Intensity Index, representing a measure of intensity of CB. The DBC-A is a reliable and internally consistent instrument with Cronbach’s alpha for the total score of *α* = .95 and for the subscales *α* ranges from .71 - .91 (Mohr et al., 2011).

#### Adaptive behaviour: Vineland Adaptive Behavioral Scale (Vineland II) and Vineland Screener

The Vineland II (Sparrow et al., 2005), administered at the first time wave, is a commonly accepted measure of adaptive skills and has been used extensively in research in subjects with intellectual disability. Data were collected through semi-structured interviews with the professional primary caretaker by certified master students and research assistants who completed training and supervision in Vineland II interviewing. A Dutch translation of the Vineland screener (Sparrow et al., 1993; van Duijn et al., 2009), a questionnaire of 90 items, each rated from 0-4, was used to assess developmental age of individuals with an estimated age between 0 and 12 years. It was administered at the second and third time wave and is a short form of the expanded interview, which covers the same four domains and age equivalents on Communication, Daily living skills, Socialization and Motor skills as in the expanded version. A combination of the four domain scores (composite score), as well as the four domain scores showed good internal consistency and reliability (Cronbach’s *α* >.95, Van Duijn et al., 2009). Since the expanded interview was only administered in the first wave, items corresponding with the screener items were selected from the expanded interview to create comparable total and domain scores of adaptive behaviour over time. A higher developmental adaptive age means more presentation of adaptive behaviour in a person.

#### Quality of life: San martin scale

A Dutch translation of the San Martin Scale (SMS) (Verdugo et al., 2014) was used to measure quality of life (QoL) and was administered on each time wave. The SMS contains 95-items, based on the QoL framework (Schalock et al., 2002), resulting in eight domains (Self-determination, Emotional Well-being, Physical Well-being, Material Well-being, Rights, Personal Development, Social Inclusion, Interpersonal Relations). Cronbach’s alpha ranges from .82 to.93 (domains) and .97 (total score) (Verdugo et al., 2014). The SMS is completed by a staff member with the most knowledge about the participant. Items are statements about the participants life, and proxies have to indicate how often the given statement occurs in the everyday life of this person on a 4-point Likert-scale, ranging from ‘never (1)’ to ‘always (4)’. Item scores result in eight QoL domain scores and a Total QoL Score, all converted to mean item scores.

#### Individual support plans

The interdisciplinary support team evaluates the individual’s support and intervention requirements and this information is formalized into an Individual Support Plan (ISP), documenting the details of the individualized support as agreed upon for a designated period, with an inclusion of the desired goals to be achieved. The ISP of each participant was evaluated and goals were analyzed for content and counted through an in-house developed scoring system with multiple scorers, based on criteria for writing support plans (Buntinx et al., 2012). Scorers received training and supervision by an licensed psychologist (Fleiss Kappa interrater reliability of .77). For each participant, goals focused on reducing CB and goals focused on developing new behaviour were counted separately.

### Statistical analysis

To test the hypothesis that group differences are present at the first wave, a multivariate analysis (MANOVA) was performed with Group (IS vs. IS+) as between subjects (BS) factor and DBC-A total (MIS) and DBC-A Intensity Index, total Adaptive behaviour score, and total QoL score as dependent variables. Significant group differences will be followed up by repeated measures analysis of variance (RM ANOVAs) with domain as within-subject (WS) factor to see whether possible group differences depended on type of domain. This would be the case when the Group x Domain interaction is significant. Consequently, only interaction effects will be reported.

Differences in distribution of mental health DSM-classifications will be evaluated with Chi-square test. Differences in total number of mental health DSM- classifications will be analyzed by a *t*-test for independent samples. To test the hypothesis that the groups differ in aspects of their individual support plan, independent samples *t*-tests were performed comparing groups on number of goals at developing new behaviour and on reducing CB, respectively.

To test the hypothesis that the groups differ in changes over time, RM ANOVAs were performed with BS factor Group, and WS factor Time, for SMS total, DBC-A total (MIS) and DBC-A Intensity Index, and Vineland scores as dependent variables, respectively. For factor Time, SIMPLE contrasts, with T2 vs T1 as first contrast and T3 vs T1 as second contrast were used to assess shorter and longer term development over time, respectively. Significant Group x Time interactions, indicating that changes over time are different for the two groups, will be followed up by RM ANOVAs with BS factor Group (IS vs IS+) and Domain as WS factor, and the *difference* score (T3 minus T1) for each domain as dependent variable, to investigate whether group differences in change over time depend on type of domain.

Data were analyzed using SPSS statistics version 28 (IBM, 2022). Significance level was set at *p*<0.05. Effect sizes were calculated using partial eta squared with η_p_^2^∼0.03 representing a weak effect, η_p_^2^∼0.06 representing a moderate effect and η_p_^2^≥0.14 representing a large effect (Cohen, 1992).

## Results

### Descriptives

The two groups, Intensive Support (IS) and extra Intensive Support (IS+) did not differ in age at assessment [*t*(102)=-1.506, *p*=.135] or in mean cognitive age equivalent [*t*(86)=.093, *p*=.926], or gender distribution, see Tables 1, 2.

**Table 1.** Characteristics for Intensive Support (IS) vs. extra Intensive Support+ (IS+).

	IS (*N* = 70)	IS+ (*N* = 35)
Age at assessment (years) (*M, SD*)	41.67 (13.87)	37.22 (14.67)
Cognitive age equivalent (months)(*M, SD*)	48.37 (36.95)	49.14 (34.38)
Gender (*N* male)	48	24

**Table 2.** Descriptives Intensive Support (IS) vs. extra Intensive Support (IS+) on measures of Challenging Behaviour, Adaptive behaviour, Quality of life and DSM-classifications. Displayed are Mean Item Scores (MIS) of total and domain scores for DBC-A (range 0-2) and SMS (range 0-4), for Vineland II age equivalents in months.

		*Intensive Support (IS) M*, *SD*	*Intensive Support+* (IS+) *M*, *SD*
Challenging Behaviour DBC-A	Disruptive	.73 (33)	1.10 (.36)
	Communication and Anxiety Dist.	.57 (.28)	.87 (.31)
	Self Absorbed	.55 (.36)	.78 (.39)
	Antisocial	.46 (.31)	.62 (.35)
	Depressive	.41 (.30)	.67 (.36)
	Social Relating	.72 (.41)	.85 (.37)
	Total	.54 (.21)	.78 (.27)
	Intensity Index	.36 (.19)	.48 (.19)
Adaptive behaviour Vineland-II	Communication	40.81 (25.62)	39.73 (25.54)
	Socialization	34.65 (25.18)	28.79 (19.17)
	Daily Living Skills	61.27 (39.60)	54.63 (33.38)
	Motor Skills	49.46 (19.13)	42.60 (23.11)
	Total average	46.28 (26.56)	42.58 (23.11)
QoL SMS	Self Determination	2.72 (.38)	2.60 (.39)
	Rights	2.97 (.36)	3.12 (.38)
	Emotional Wellbeing	2.93 (.44)	3.14 (.42)
	Social Inclusion	2.90 (.41)	2.93 (.45)
	Personal Development	3.33 (.30)	3.20 (.39)
	Interpersonal Relationships	2.57 (.43)	2.72 (.49)
	Material Wellbeing	2.23 (.49)	2.17 (.43)
	Physical wellbeing	2.87 (.40)	2.90 (.42)
	Total	2.82 (.28)	2.85 (.31)
DSM	Presence of mental health) classification (other than intellectual disability) *N, %*	42 (60.9%)	28 (82.4%)
	No. of mental health classification (other dan intellectual disability)*	1.40 (.73)	1.39 (.57)
	ASD classification %	76%	75%

* NB: only for those participants who received a DSM classification.

### Challenging behaviour, adaptive behaviour and quality of life

The MANOVA on group differences resulted in a significant multivariate effect, [*F*[4,94)=7.884, *p*<.001, η_p_^2^=.251]. The between-subjects effects were significant for DBC-A total (MIS), *F* (1,97)=26.454, *p*<.001, η_p_^2^=.214, and the DBC-A Intensity Index, *F* (1,97)=9.049, *p*=.003, η_p_^2^=.085, but not for adaptive behaviour (Vineland, *p=*.383), and QoL (SMS, *p=*.556). Repeated measures analyses for DBC-A subdomain scores showed a nonsignificant Group* Domain interaction (*p*=.077) indicating that group differences did not depend on type of domain. Post hoc analyses revealed that group differences were significantly higher for IS+ for all subdomains (.027>p<.001), except Social Relating (p=.107).

Individuals in the IS+ group more often had one or more DSM mental health classification (other than intellectual disability), compared to the IS group, χ_2_ (1, *N*=103)=4.828, *p=*.028). When a DSM classification is present, the IS+ group did not differ in total number of DSM mental health classifications from the IS group (*p*=.942), nor did the proportion of Autism Spectrum Disorder classifications (*p*=.909).

### Individual support plans

Table 3 summarizes ISP outcomes. On number of goals targeted at development of new behaviour, groups did not differ significantly (*p*=.612), but the IS+ group showed a significantly higher number of goals targeted at reducing challenging behaviour [*t*(96)=2.237, *p*=.028, *d*=2.969].

**Table 3.** Individual support plans for Intensive Support (IS) vs. Intensive Support+ (IS+).

	*IS*	*IS+*
No. of goals targeted at development of new behaviour	1.19 (.20)	1.36 (.29)
No. of goals targeted at reducing CB	3.31 (.37)	4.73 (.52)

### Outcome of longitudinal follow-up

#### Challenging behaviour

**Figure 1.**
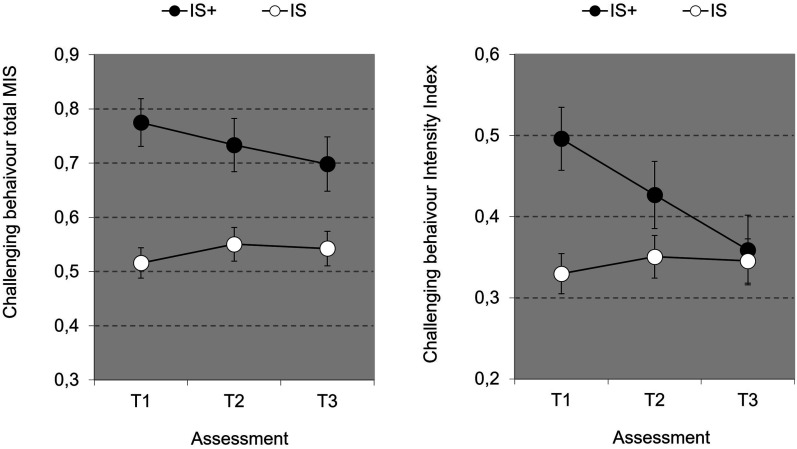
Challenging behaviour over time as a function of Group with DBC-A total score (MIS) (± SEM)(left graph) and DBC-A (Intensity Index) (± SEM)(right graph).

**Figure 2.**
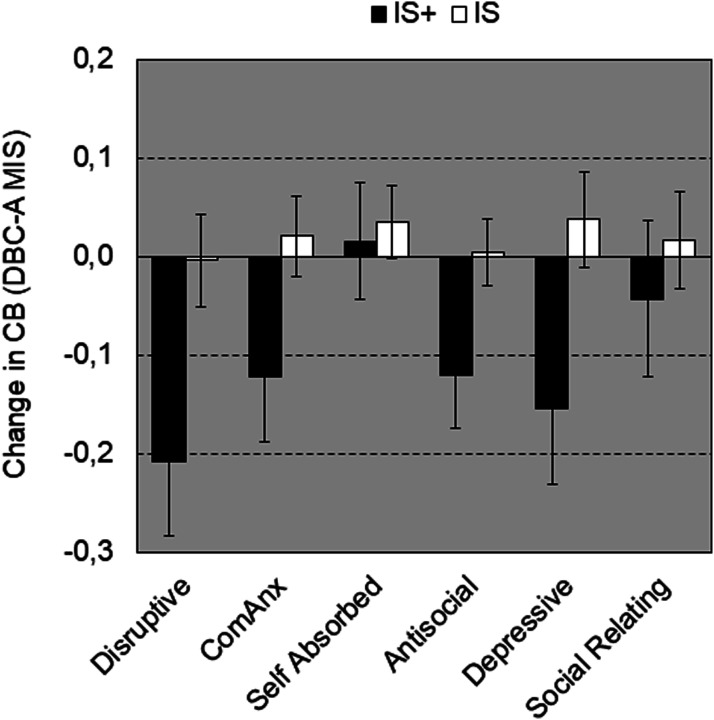
Change over time (T3 vs. T1) ± SEM in challenging behaviour total score (MIS) as a function of Challenging Behaviour domain and Group. Negative change in CB means decrease in CB over time.

For DBC-A total (MIS), main effect of Time was not significant (*p*=.384). Also the interaction Time*Group was not significant for contrast 1 (p=.115) and contrast 2 (p=.06). For DBC-A Intensity Index, the main effect Time was not significant (p=.06), but the Time*Group interaction was significant [*F*(2,70)=4.204, *p*=.019, η_p_^2^=.107], accompanied by a nonsignificant first contrast (*p*=.06) and a significant second contrast (*p*=.005, η_p_^2^=.105), indicating that decrease in intensity over time was only present in the IS+ group (see Figure 1). Repeated measures analyses for DBC subdomain scores show a nonsignificant Group*Domain interaction (*p*=.20) which indicates that group differences for contrast 2 did not depend on type of domain (Figure 2.)

#### Adaptive behaviour

**Figure 3.**
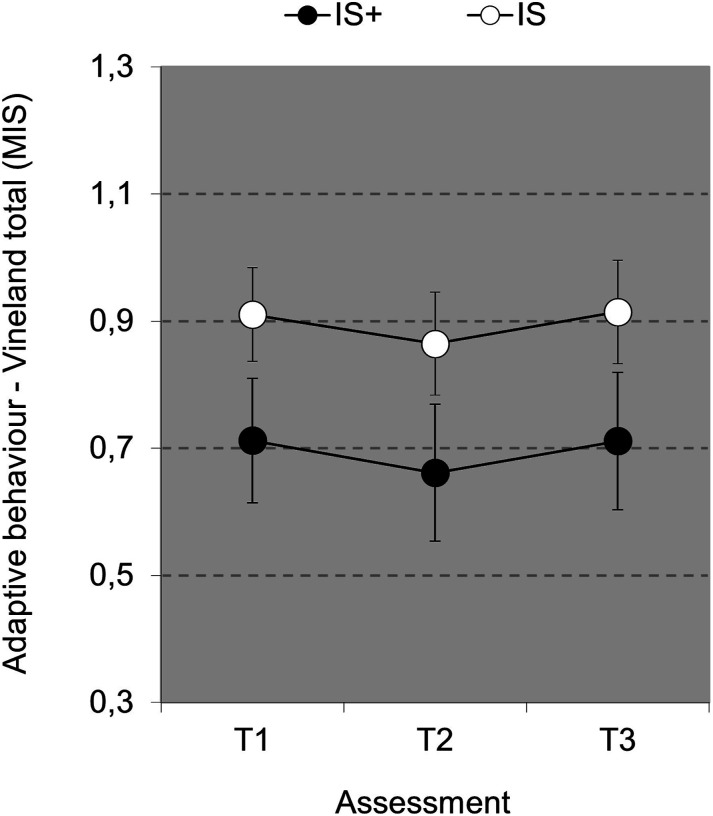
Overall Adaptive Behaviour (± SEM) Score (MIS) over time as a function of Group.

Total adaptive behaviour shows a significant main effect for Time [*F*(2,112)=3.851, *p*=.024, η_p_^2^=.064], further specified by a significant first contrast (T1 vs. T2), [F(1,56)=5.197, *p*=.026, η_p_^2^=.085], reflecting a decrease in level of adaptive behaviour at T2, and a nonsignificant second contrast (T1 vs. T3), (*p*=.94), reflecting that at T3 adaptive behaviour recovered to the level at T1 (see Figure 3). The Group*Time interaction, overall and per contrast, was not significant (.91<*p*<.99). The main effect of Group was not significant (*p*=.123).

#### Quality of life

**Figure 4.**
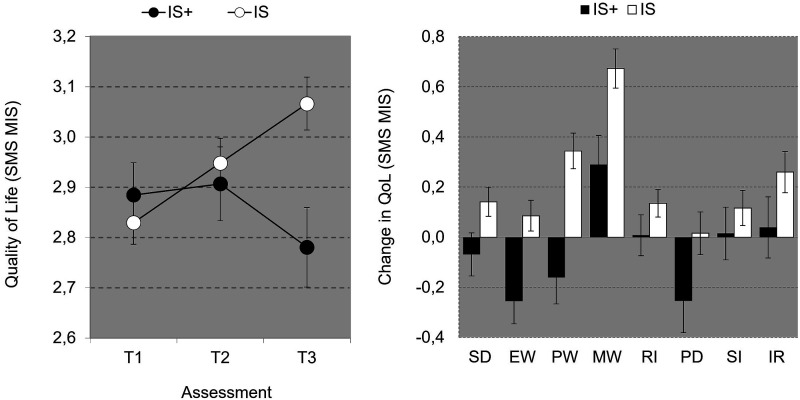
Overall Quality of Life (San Martin Scale Mean Item Score ± SE) over time as a function of Group (left graph) and changes in Quality of Life (± SE) as a function of Quality of Life domain and Group (right graph). Domains right graph: Self Determination (SD), Emotional Wellbeing (EW), Physical Wellbeing PW), Material Wellbeing (MW), Rights RI), Personal Development (PD), Social Inclusion (SI), Interpersonal Relationships (IR).

Total Quality of Life (SMS, Figure 4) shows a nonsignificant main effect for Time (*p*=.196), but a significant Time*Group Interaction [*F*(2,62)=6.177, *p*=.004, η_p_^2^=.166], accompanied by a nonsignificant first contrast (p=.248), and a significant second contrast [*F*(1,63)=12.394, *p*<.001, η_p_^2^=.164], which is accounted for by an increase over time in QoL for the IS group (see Figure 4). Post hoc RM analyses for T3 vs. T1 confirmed a significant increase in QoL over time for the IS group [*F*(1,55)=23.245, *p*<.001, η_p_^2^=.297] but not for the IS+ group (p=.540).

Repeated measures analyses for SMS subdomain difference scores (T3 vs. T1) show no significant Group*Domain interaction (p=.091) which indicates that group differences in changes over time (T3 vs. T1) did not depend on type of domain (Figure 4). Visual inspection of differences scores per domains suggests that only SMS domain Material Wellbeing showed an improvement over time for the IS+ group. Post-hoc RM-analysis for this domain confirmed this suggestion [*F*(1,24)=4.208, *p*=.051, η_p_^2^=.149].

## Discussion

This study aimed to explore the efficacy of the provided support over a period of three years and explored factors that help to decide which persons with intellectual disability and severe challenging behaviour should be entitled to receive extra intensive support. A group of individuals who received extra intensive support (IS+) were compared with a group of individuals who received intensive support (IS) as usual.

### Efficacy of treatment

A strength of the study is its longitudinal design, providing valuable insights in potential treatment-related changes in behaviour, adaptive functioning and quality of life over three years’ time of individuals receiving intensive or extra intensive support. There were no significant changes in total level of challenging behaviour over time for both the intensive support and the extra intensive support group. However, intensity of challenging behaviour was found to decrease in the extra intensive support group.

Adaptive behaviour did not improve over time for both groups. Quality of life improved in the intensive support group but not in the extra intensive support group, which could have been due to the different focus of care. In the extra intensive support group, focus was mostly on management of challenging behaviour, i.e. an emphasis on managing problematic behaviour rather than on improving development, particularly for individuals who receive extra intensive support. This is in line with for example Unwin et al. (2017) who found an overreliance on medication and contact time with psychiatrists, and relatively less attention for help by clinical psychologists, physiotherapists, and community nurses for the management of challenging behaviour. Extra intensive support is mostly used to implement a higher staff-individual ratio. Bruinsma et al. (2022) found that more staff members working per person with intellectual disability (higher staff-individual ratio) was related with lower quality of life scores in persons with intellectual disability and challenging behaviour, especially for social inclusion and self-determination. In line with our findings, it is therefore conceivable that that the intensity of being continuously monitored in very complex care (individual staff ratio 1:1 or even 1:2) could be experienced as more restrictive, which could affect quality of life negatively. Another explanation of the extra intensive support group not improving in quality of life might be that in cases of severe challenging behaviour, a longer time period is needed to improve quality of life.

Improvement in challenging behaviour in the extra intensive support group over time was associated with more intensive individual care, but the professional’s ability to create a different environment or support setting through higher availability of financial means might also be an important contributor. It is known that not only physical, but also organizational aspects of the onto-, micro-, meso-, exo- and macrosystem may be of influence on the incidence of challenging behaviour (Bronfenbrenner and Morris, 2007; Olivier-Pijpers et al., 2018; Olivier-Pijpers et al., 2020). For example, the relevance of direct physical environment as earlier addressed by Roos et al. (2022), but also factors such as grouping of residents can be of influence on challenging behaviour (Olivier-Pijpers et al., 2020). Furthermore, factors within staff are important to consider, since individuals with extra intensive support are highly dependent upon them (Wissink, 2015; Embregts et al., 2023). For example as staff turnover or staff’s sense of safety (Olivier-Pijpers et al., 2020). In future research on the effects of extra intensive support for a group with specific needs, it could be important to analyze the contribution of environmental and organizational factors to incidence and severity of challenging behaviour and quality of life in addition to more intensive individual support.

### Differences between groups receiving IS or IS+ at time of inclusion

As expected, the individuals with extra intensive support showed higher levels of challenging behaviour, and their challenging behaviour is experienced as more severe. Both groups showed a similar profile of challenging behaviour, i.e. differences between groups could not be pinpointed to one or more specific domains of challenging behaviour. Furthermore, individuals with extra intensive support showed a higher number of co-morbid mental health classifications and a stronger focus on goals in ISPs to reduce challenging behaviour than on goals to develop new behaviour.

The finding that challenging behaviour is initially higher in the extra intensive support group is partly in line with the findings of Tevis (2020), who argues that intensity and frequency of aggression against others and self is the most important factor associated with direct (more staff) and indirect needs, such as additional training to maintain safety, supervision and clinical overview. Our results indicate that not only level of disruptive behaviour, but also levels of other forms of challenging behaviour, including internalizing challenging behaviour, were higher in the IS+ group.

Regarding adaptive behaviour, no initial differences were found between the groups, and this result was independent of type of adaptive behaviour. Level of adaptive behaviour does not seem to differentiate in support needs in our group which is in line with the results of Buntinx et al. (2010a). For quality of life, also no initial differences were found between groups.

### Limitations

It is virtually impossible to account for all potentially influential factors in this highly complex group. For example, variables representing more complex medical needs were not included and therefore possible interference of severity of medical conditions cannot be evaluated. Indeed, excluding people with mostly medical needs, only people with intellectual disability for whom challenging behaviour was the most important support need were selected. Furthermore, by matching the intensive support and extra intensive support groups on gender, age and cognitive functioning, variability due to this matching might have been missed. Since severe challenging behaviour is known to occur more often in the lower range of cognitive functioning, the group on this end of this spectrum might be relatively overrepresented in the intensive support sample. Another limitation is the absence of medication data in this study, as medication could influence level of behaviour. Pharmacological interventions in adults with intellectual disability are often administered, especially when challenging behaviour is present for the regulation of behaviour. Their potential influences could be diverse (Bowring et al., 2017; Groves et al., 2023).

Unavoidably, the last time wave was partly affected by covid measures, which complicates the interpretation of its results. In most qualitative studies it is hypothesized that people with an intellectual disability and high support needs display more anxiety, distress, or challenging behaviour during quarantine periods, because of their already increased vulnerability, accompanied by sudden changes in daily routine and social interaction (Buonaguro and Bertelli, 2021; Doody and Keenan, 2021). Consequently, in our study an increase in challenging behaviour was expected instead of the observed decrease. It is therefore possible that the improvement in challenging behaviour in the extra intensive support group is an underestimation.

Due to the level of intellectual disability, self-evaluation was not feasible. Therefore, proxy assessment by primary staff was used to complete the questionnaires regarding challenging behaviour, adaptive functioning and quality of life, probably increasing likelihood of reporting socially desirable answers or underestimation of quality of life of adults with intellectual disability, compared with self-reports, especially on more subjective aspects as emotional/psychological domains (Koch et al., 2015). The use of multiple-proxy assessment may help to increase reliability. Future research can be improved by using structured observations in addition to questionnaires, to serve the ecological validity of findings.

In order to strengthen the generalizability of our conclusions, four residential disability service organizations were involved. These organizations offered similar type of group home living, and are highly specialized in care for people with intellectual disability and (severe) challenging behaviour. Therefore, one should not generalize the results in these highly specialized and organized organizations to smaller organizations with less specialization.

### Clinical implications

This research contributes to knowledge to correctly and responsibly allocate funding to enable extra intensive support, and its effectiveness over time. It appears that those individuals with more severe challenging behaviour and more mental health problems are the ones receiving extra intensive support, i.e. serving those who need this support most. Following the results of this study it is furthermore recommended to develop allocation policies for extra intensive support. An objective assessment framework of measures of challenging behaviour, adaptive behaviour and quality of life, also over time, should be implemented as an important and valuable addition to qualitative descriptions as evaluation method. Measures such as the Supports Intensity Scale (Thompson et al., 2004) show promising results in identifying those in need of extra intensive support - for example (Wehmeyer et al., 2009).

Results of this study indicate the efficacy of extra intensive support, focused on reducing challenging behaviour as an initial intervention. Diminishing challenging behaviour to restore stability can be a primary emphasis in clinical practice. However, once this goal has been achieved, renewed emphasis should be placed on the restoration of quality of life. Our results indicate that reducing challenging behaviour by extra intensive support does not necessarily coincides with an enhancement in quality of life.

The evaluation whether individuals with intellectual disability and severe challenging behaviour are entitled to extra intensive support, is currently mainly oriented on client characteristics (onto-system), but should shift towards a more integrated approach of understanding the complexity of challenging behaviour, taking into account contextual factors as well. The presence of challenging behaviour should be seen as a starting point in this evaluation, recognizing the interactive interplay of different factors of individual functioning, as outlined by Embregts et al. (2023). This paper moreover adds a heightened emphasis on systematic, longitudinal monitoring, especially within clinical practice.

In conclusion, this study demonstrated the effectiveness of extra intensive support in a three years follow-up. Furthermore, it was shown that extra intensive support treatment was indeed offered to participants presenting with the most severe level of challenging behaviour in combination with more mental health classifications. Differences in challenging behaviour between groups could not be pinpointed to specific domains of challenging behaviour. In addition, extra intensive support was also characterized by a higher number of support plan goals. The results suggest that the governmental funding policies and systems of service provision in the country are effectively meeting the needs of those requiring this extra high level of support. Furthermore, more intensive support resulted in a reduction in level of challenging behaviour over three years’ time, underscoring the effectiveness of the implemented extra care.
